# Detection of Superoxide Anion Oxygen Reduction Reaction Intermediate on Pt(111) by Infrared Reflection Absorption Spectroscopy in Neutral pH Conditions

**DOI:** 10.1021/acs.jpclett.0c03510

**Published:** 2021-02-04

**Authors:** Valentín Briega-Martos, William Cheuquepán, Juan M. Feliu

**Affiliations:** † Instituto de Electroquímica, Universidad de Alicante, Apdo. 99, E-03080 Alicante,Spain

## Abstract

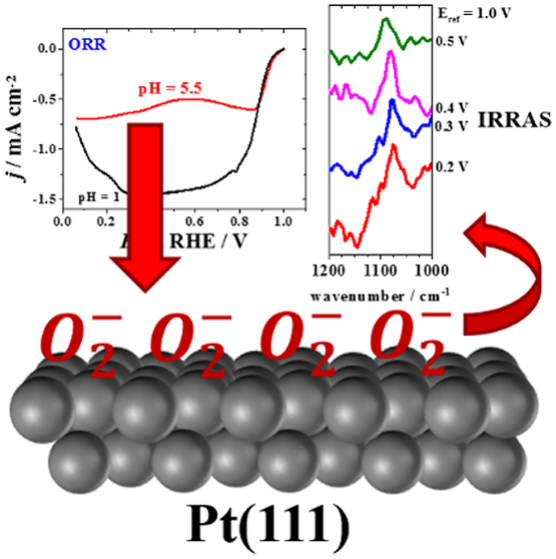

In this work, *in situ* external infrared reflection absorption spectroscopy (IRRAS) is successfully employed for the detection of intermediate species in the oxygen reduction reaction (ORR) mechanism on a flat and well-defined Pt surface. Superoxide anion species (O_2_^–^) are detected on the Pt(111) surface in an O_2_-saturated solution with a NaF/HClO_4_ mixture with pH 5.5 by the observation of a O–O vibration band at *ca.* 1080 cm^–1^. The observation of O_2_^–^ without the use of any other additional method of signal enhancement is possible because in these experimental conditions O_2_^–^ is the main ORR-generated intermediate and its reactivity is limited in this pH. This leads to the accumulation of O_2_^–^ near the Pt surface, facilitating its identification.

The oxygen reduction reaction (ORR) is the electrocatalytic reaction that takes place in the cathode of proton-exchange membrane fuel cells (PEMFCs), the efficiency and cleanliness of which make them one of the most promising technologies for distributed power generators and electric vehicles. The sluggish kinetics of the ORR even for Pt, the best pure metal for this reaction, is one of the main drawbacks of fuel cell technology and hinders its commercialization.^1,2^ Hence, it is necessary to develop new electrode materials with improved electrocatalytic activity toward the ORR and high durability in order to make this technology suitable for commercial uses. In order to achieve this goal, a deep knowledge of the ORR mechanism obtained through fundamental studies is essential for the rational development of new electrocatalysts suitable for practical electrodes.

The ORR is a complex electrochemical reaction than requires the transfer of four electrons to yield water as the final product. As a consequence, several different intermediates can take part in the mechanism of the ORR, such as O_ads_, OH_ads_, HO_2,ads_/HO_2_^•^, O_2,ads_^–^/O_2_^–^, H_2_O_2,ads_/H_2_O_2_ or HO_2_^–^/HO_2,ads_^−^.^3^ The high number of previous computational and experimental studies about the ORR evidence that the reaction occurs through a set of parallel reactions with a similar rate. Within this reaction mechanism, small changes in the experimental conditions could modify the relative rates of the different parallel steps, giving rise to a significant change in the global reaction mechanism.^4^ Therefore, it is mandatory to experimentally identify the nature of the ORR intermediate species in order to unambiguously establish which reaction mechanism is taking place at certain experimental conditions. However, the short lifetime and the low coverage of most of the intermediates, as well as the possible influence by other coadsorbed species,^5^ make necessary the use of new experimental techniques and strategies with very high surface sensitivity.

Apart from the use of the rotating ring-disk electrode (RRDE) that has allowed detecting H_2_O_2_ as a final product of the ORR under different conditions,^6,7^ several spectroscopic techniques with special configurations have been employed in order to identify other reaction intermediates during the last 15 years. Teliska *et al*. assigned the oxygen adsorption sites for O and OH species by near-edge X-ray adsorption spectroscopy (XANES) in combination with *ab initio* theoretical calculations in ORR conditions.^8^ Shao *et al*. identified experimentally for the first time O_2,ads_^–^ as an ORR intermediate in alkaline media at pH 11 on a Pt thin film by surface-enhanced infrared reflection absorption spectroscopy with attenuated total reflection (ATR-SEIRAS).^9^ Friebel *et al*. were able to differentiate the chemisorbed oxygen-containing species on Pt from the different platinum oxides by XANES.^10^ Casalongue *et al*. observed using ambient pressure X-ray photoelectron spectroscopy (APXPS) that during the ORR on a fuel cell cathode composed of Pt nanoparticles the nonhydrated OH species are the dominant species, and with the assistance of DFT calculations they showed that the reduction of these species requires less overpotential than that of hydrated OH.^11^ More recently, the HO_2,ads_ intermediate has been identified on a Pt(111) single-crystal electrode in 0.1 M HClO_4_ by shell-isolated nanoparticle-enhanced Raman spectroscopy (SHINERS), while O_2,ads_^–^ was detected on the three Pt basal planes in alkaline solution at pH 10.^12^ In the same year, Nayak *et al*. observed by multibounce attenuated total reflection infrared (ATR-IR) spectroscopy bands attributed to HO_2,ads_ and H_2_O_2_,_ads_ on Pt nanoparticles in 0.1 M HClO_4_.^13^ HO_2_^•^ soluble species was previously suggested by Gómez-Marín *et al*. as an intermediate in the ORR mechanism by cyclic voltammetry on Pt(111) and polycrystalline Pt in 0.1 M HClO_4_ varying different experimental conditions.^4,14^ The existence of HO_2_^•^ intermediate as a bifurcation point in the ORR mechanism was proposed by Staszak-Jirkovský *et al*. from experiments with Au–Pd^15^ and by Ruvinskiy *et al*. by experiments with Pt nanoparticles supported on different carbon nanomaterials.^16^ ORR and hydrogen peroxide reduction reaction (HPRR) cyclic voltammetry experiments on different Pt single crystals in solutions with pH values ranging from acidic to neutral values in the absence of anion-specific adsorption also suggest that HO_2_^•^ constitutes a bifurcation point in the ORR mechanism previous to the formation of H_2_O_2_.^17−20^ The latest works presenting spectroscopic evidence of ORR intermediates comprise the work by Dong *et al*. in which OOH_ads_ and OH_ads_ Raman bands are observed with different intensity on Pt(211) and Pt(311) surfaces in 0.1 M HClO_4_ by the SHINERS technique^21^ and the work by Kukunuri and Noguchi, who attribute to the O_2,ads_^–^ intermediate an infrared band observed at *ca.* 1100 cm^–1^ in 0.1 M HClO_4_ by SEIRAS with a Pt/Au/ZnSe surface and spectral analysis under O-isotope and D_2_O conditions.^22^

In the present work, *in situ* external infrared reflection absorption spectroscopy (IRRAS) in a NaF/HClO_4_ mixture solution with pH 5.5 is employed to identify O_2,ads_^–^ intermediate on Pt(111). These pH conditions and the absence of anion adsorption have allowed the detection of an ORR intermediate on a flat surface by infrared spectroscopy without the use of any other system to enhance the spectroscopic signal.

Figure 1 shows the voltammetric results for the ORR on Pt(111) in 0.1 M HClO_4_ and in a NaF/HClO_4_ mixture with pH 5.5 in both hydrodynamic conditions with a rotation rate of 2500 rpm (panel A) and in nonhydrodynamic conditions (panel B). On the one hand, an improvement in the current density near the onset potential can be observed for pH 5.5, in agreement with previous results.^17,18^ On the other hand, the main difference between both pH values is the diminution of the limiting current density (*j*_lim_) in the case of pH 5.5. This was also observed in the previous works and was attributed to the existence of a bifurcation point in the ORR mechanism before H_2_O_2_ formation,^17−19^ because this diminution of *j*_lim_ is not observed when the HPRR is investigated in the same conditions.^23^ The decrease of *j*_lim_ for pH 5.5 was explained in terms of the inability for O_2_H^•^ intermediate, or its basic form, O_2_^–^, to react at the interface and its necessary diffusion to the bulk solution, with the subsequent loss of Faradaic efficiency. The fact that this decrease is observed only for pH > 3 and is larger as the pH increases was rationalized by considering that, below the p*K*_a_ value of 4.8 for the O_2_H^•^/ O_2_^–^ equilibrium,^24^ the O_2_H^•^ species would be the main species and it would be easily reduced, while at higher pH values the major species should be O_2_^–^, which could not be easily reduced and would diffuse to the bulk solution. The shape of the polarization curve is different in the absence of hydrodynamic conditions because in this case the concentration of the reactant, O_2_, is not well-maintained near the surface. In the case of pH 5.5, a small peak or overshooting is observed at high potentials because, added to the fact that in pH 5.5 the limiting current densities are much lower, in this case the kinetic activity is higher than at pH 1.1.^17^ Moreover, there is a progressive slight increase of the current density as the potential is swept to more negative values for both pH values. The remarkable consequence from the different electrochemical behavior at neutral pH in the absence of anion-specific adsorption is that it could lead to differences in the concentration of the ORR intermediates near the Pt surface, and therefore, it is interesting to perform *in situ* infrared spectroscopy measurements in these experimental conditions.

**Figure 1:**
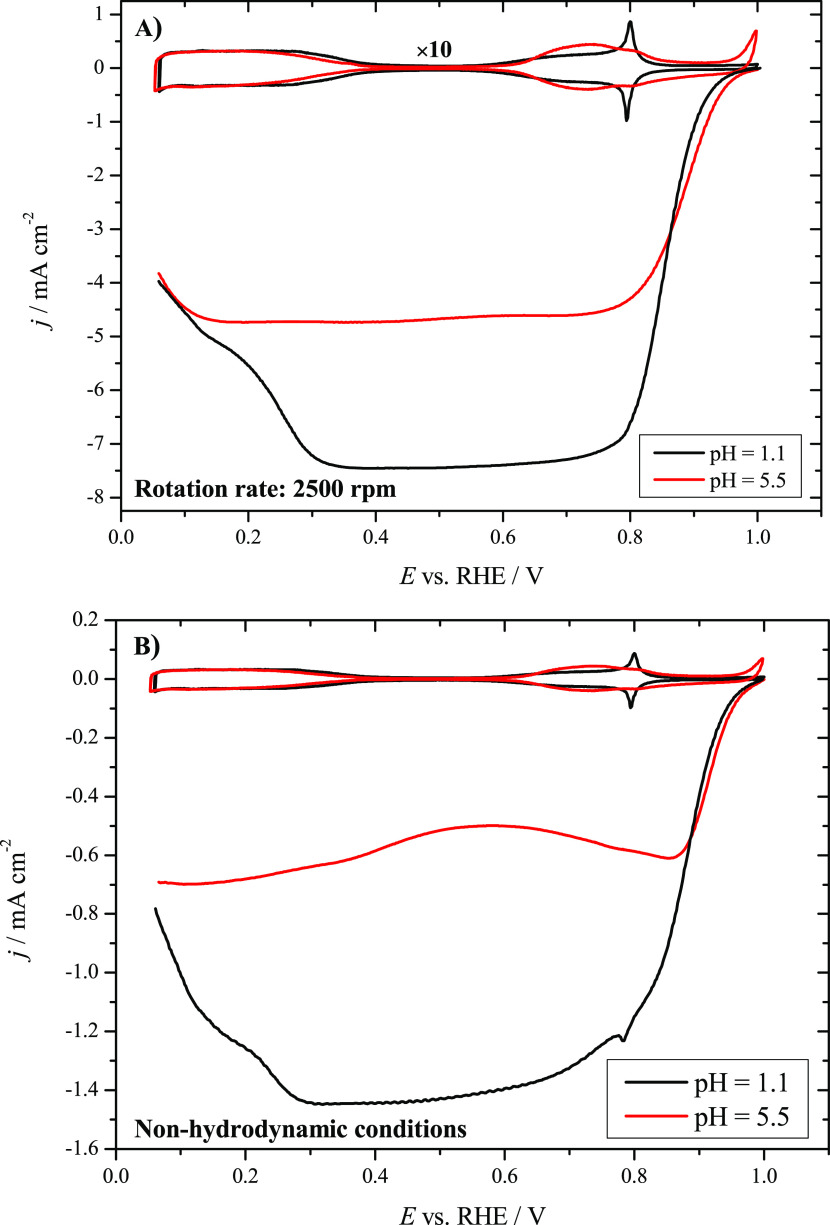
Polarization curves for the ORR on Pt(111) in O_2_-saturated 0.1 M HClO_4_ (black line) and in O_2_-saturated NaF/HClO_4_ mixture solution with pH 5.5 (red line) with a rotation rate of 2500 rpm (A) and in nonhydrodynamic conditions (B). The cyclic voltammograms in absence of O_2_ are also included. Scan rate: 50 mV s^–1^.

The electrochemical interface between the Pt(111) surface and the NaF/HClO_4_ solution with pH 5.5 has been studied by *in situ* IRRAS in ORR conditions. The resulting infrared spectra presented in Figure 2 show the appearance of a band at *ca.* 1080 cm^–1^ from 0.5 V vs RHE to 0.2 V vs RHE. Nakamura *et al*. noted a band at *ca.* 1080 cm^–1^^25,26^ or *ca.* 1050 cm^–1^^27^ by IRRAS measurements on Pt(111) in a deoxygenated 0.1 M NaF solution, and they ascribed this band to adsorbed OH. We have carried out measurements in the same conditions as in Figure 2 but in the absence of dissolved O_2_, and no band was observed in the region around 1080 cm^–1^ (see Figure S1 in the Supporting Information). In addition, in the works by Nakamura *et al*., the band assigned to adsorbed OH is already observable at high potentials (1.0 V vs RHE), and it is observed only down to 0.6 V in the case of Pt(111).^25−27^ However, in the present work the band appears from 0.5 to 0.2 V vs RHE. Therefore, the intermediate species responsible of the band at 1080 cm^–1^ observed in this work should be different than adsorbed OH. A band at *ca.* 1095 cm^–1^ was observed by Kukunuri *et al*. from SEIRAS measurements on a Pt/Au/ZnSe in 0.1 M HClO_4_.^22^ With the aid of additional experiments in O-isotope conditions, they ascribed this band to the O–O stretching vibration of O_2,ads_^–^ intermediate species. They explain the possibility of detecting this intermediate because of the local pH near the electrode, which in their conditions would be maintained above the p*K*_a_ of O_2_H^•^/ O_2_^–^. In this way, the lifetime of O_2_^–^ near the Pt surface increases at potentials above 0.7 V vs RHE would increase, allowing its detection. In the previous work using SHINERS to detect the ORR intermediates in alkaline media, a band centered at *ca.* 1150 cm^–1^ was observed for the three Pt basal planes, and a calculated frequency of 1182 cm^–1^ was obtained from DFT calculations.^12^ The value for the observed band in this work is close to these wavenumbers, and the little differences could be explained by differences in the coverage of O_2_^–^ intermediate or the different surface charge because the difference between the studied pH values (5.5 and 10.3) is important. In our work, because of the use of NaF buffer at pH 5.5, the formation of O_2_H^•^ is negligible in front O_2_^–^. In addition, because a buffer solution is used, the local pH near the surface is better maintained, close to the nominal value of the bulk solution. In these conditions the reduction of O_2_^–^ would be inhibited at the whole studied potential range because the measured current density is practically half of the current density measured for pH 1.1, as inferred from Figure 1. Therefore, the apparition of the band at *ca.* 1080 cm^–1^ could be related to O_2_^–^ and its lower reactivity in these conditions, which would favor the accumulation of O_2_^–^ intermediate at the Pt surface. The fact that this band appears noticeably from 0.5 V vs RHE to more negative values could be due to the lack of controlled hydrodynamic conditions in the IRRAS setup, because the O_2_ concentration near the surface would be low and an additional overpotential would be needed to generate the required surface concentration of the detected intermediate (see the comments above regarding Figure 1B). In conclusion, the detection of O_2_^–^ in the flat surface of the Pt single-crystal electrode by IRRAS spectroscopy in these conditions is possible because of the predominance of O_2_^–^ intermediate and its limited reactivity at pH 5.5, which increase its lifetime at the Pt surface.

**Figure 2:**
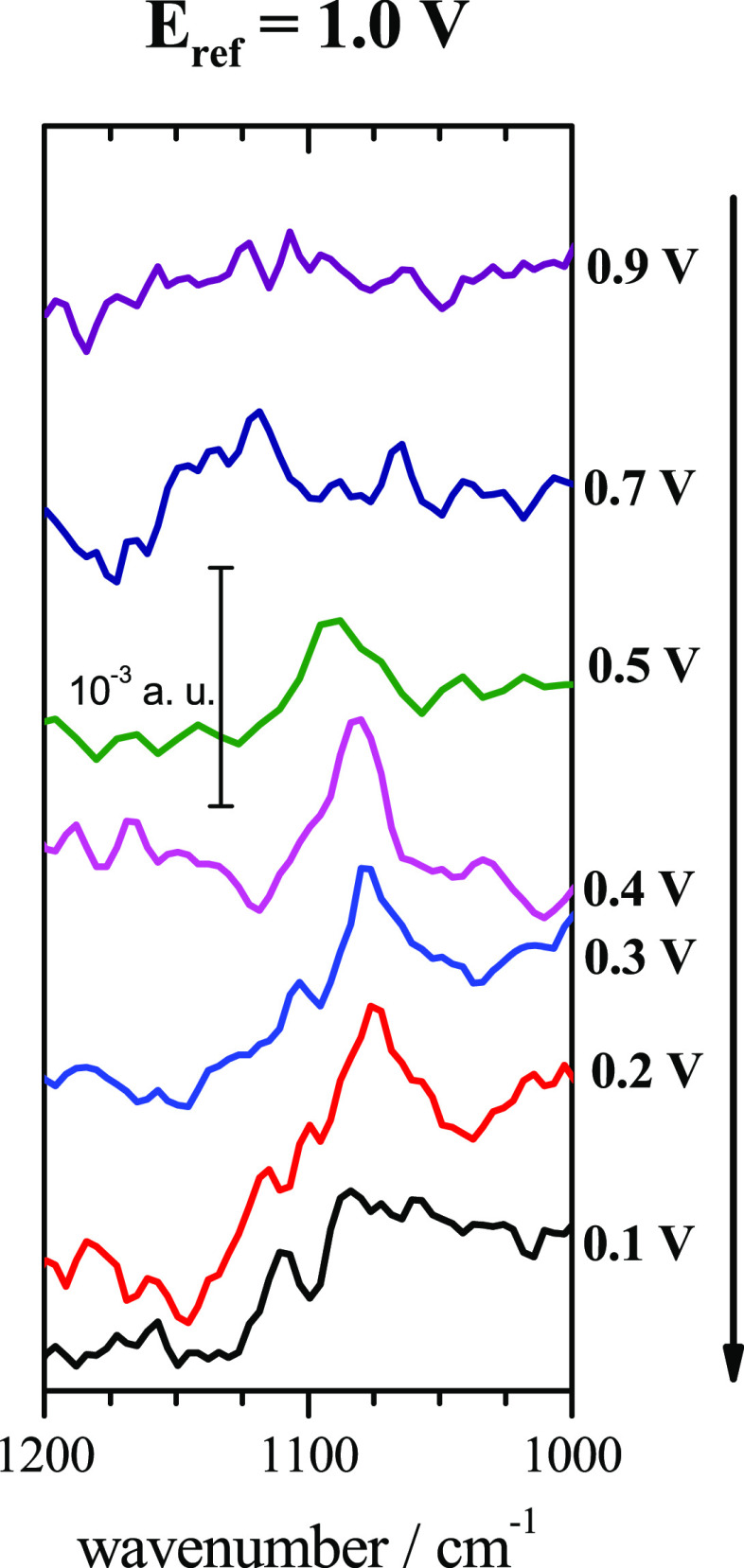
*In situ* FT-IR spectra for Pt(111) in O_2_-saturated NaF/HClO_4_ mixture solution with pH 5.5. Number of interferograms, 30; resolution, 4 cm^–1^.

In order to discard that the observed band corresponds to H_2_O_2_ species, *in situ* IRRAS experiments for Pt(111) in the same solution with pH 5.5 saturated in Ar and in the presence of 1.7 mM H_2_O_2_ were carried out. The spectra in Figure 3 show the appearance of a negative band at *ca.* 1150 cm^–1^ when potential is swept from positive to negative values. This band corresponds to the O–O vibration for H_2_O_2_, which is consumed as the potential is increased, and therefore, the band observed in ORR conditions cannot be ascribed to the H_2_O_2_ species, because it is located at *ca.* 1080 cm^–1^.

**Figure 3:**
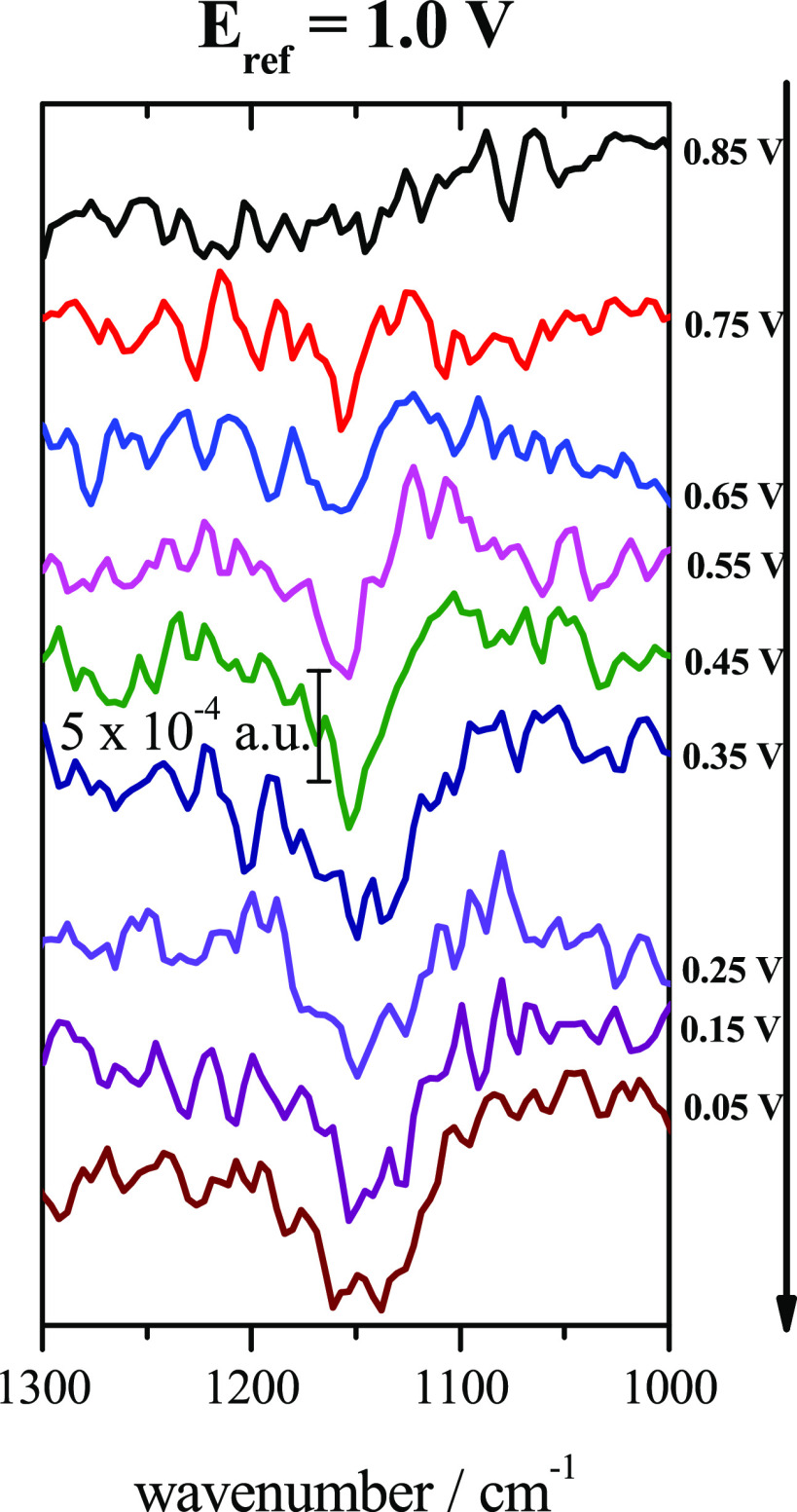
*In situ* FT-IR spectra for Pt(111) in a NaF/HClO_4_ mixture solution with pH 5.5 and 1.7 mM H_2_O_2_. Number of interferograms, 30; resolution, 4 cm^–1^.

To summarize, one of the ORR intermediates, O_2,ads_^–^, has been identified for the first time without using any signal enhancement configuration on Pt(111) by means of an infrared spectroscopy. This has been achieved because for the selected working solution, a NaF/HClO_4_ mixture with pH 5.5, the pH near the Pt surface is buffered at a value in which O_2_^–^ is the predominant intermediate and cannot be further reduced. Therefore, O_2_^–^ species can accumulate at the electrode surface, allowing its detection by the IRRAS technique. This study opens the door to new strategies that can facilitate the detection of ORR intermediate on well-defined surfaces in order to obtain new information about the mechanism of this reaction.

## Experimental Methods

The single-crystal electrode with Pt(111) well-defined orientation was prepared from a Pt bead *ca.* 6 mm in diameter and cleaned according to the methodology described by Clavilier *et al*.^28,29^ The counter electrode and reference electrode were in both electrochemical and spectroelectrochemical measurements a Pt electrode cleaned by flame-annealing and a Ag/AgCl, KCl (saturated) electrode in contact with the working solution through a Luggin capillary. All potential values have been converted into the RHE scale.

Electrochemical experiments were carried out in the hanging meniscus configuration following the general procedure described in ref (30). The *in situ* infrared spectroscopy experiments were carried out in the thin-layer configuration according to the IRRAS methodology described in refs (31) and (32) using a BaF_2_ window and employing the subtractively normalized interfacial Fourier transform infrared reflection spectroscopy (SNIFTIRS) procedure.^33−35^ Further experimental details are described in the Supporting Information.

## Supporting Information Available

Detailed experimental procedures and IRRAS measurements in absence of O_2_ (PDF)
