# Supplementary material for: Detection of Superoxide Anion Oxygen Reduction Reaction Intermediate on Pt(111) by Infrared Reflection Absorption Spectroscopy in Neutral pH Conditions
Source: J Phys Chem Lett. 2021 Feb 4;12(6):1588–92. doi: 10.1021/acs.jpclett.0c03510 (PMC8460065; doi:10.1021/acs.jpclett.0c03510)
Supplement: Supplementary file 1 — jz0c03510_si_001.pdf [file jz0c03510_si_001.pdf]

SUPPORTING INFORMATION

Detection of Superoxide Anion Oxygen Reduction Reaction Intermediate on Pt(111) by Infrared Reflection Absorption Spectroscopy in Neutral pH Conditions

*Valentín Briega-Martos,[†] William Cheuquepán,[†,‡] Juan. M. Feliu[†,]**

[†]Instituto de Electroquímica, Universidad de Alicante, Apdo. 99, E-03080 Alicante, Spain

AUTHOR INFORMATION

Corresponding Author

*(J.M.F.) Instituto de Electroquímica, Universidad de Alicante, Apdo. 99, E-03080 Alicante, Spain. E-mail: juan.feliu@ua.es.

DETAILED EXPERIMENTAL PROCEDURES

The single-crystal electrode with Pt(111) well-defined orientation was prepared from a Pt bead ca. 6 mm in diameter and cleaned according to the methodology described by Clavilier et al.[1,2] The

counter electrode and reference electrode were in both electrochemical and spectroelectrochemical measurements a Pt electrode cleaned by flame-annealing and a Ag/AgCl, KCl (saturated) electrode in contact with the working solution through a Luggin capillary. All potential values have been converted into the RHE scale.

The working solutions were prepared using concentrated $HClO_4$ (Merck, for analysis), NaF (Merck, Suprapur, 99.99%) and ≥30% (RT) H_2O_2 (Fluka, *Trace*SELECT® Ultra, for trace analysis). Ar and O_2 (N50) were also employed. Ultrapure water (Elga PureLab Ultra, 18.2 MΩ cm) was used for glassware cleaning and preparing the solutions.

Electrochemical experiments were carried out in the hanging meniscus configuration following the general procedure described in ref. 3. A signal generator EG&G PARC and eDAQ EA 161 potentiostat with an eDAQ e-corder ED401 recording system were used for the (spectro)electrochemical experiments. ORR measurements in hydrodynamic conditions were carried out in the hanging meniscus rotating disk electrode (HMRDE) using an EDI101 rotating electrode system and rotation rate was controlled by a CTV 101 unit (both Radiometer Analytical). In-situ IRRAS measurements were performed using a Nexus 8700 (Thermo Scientific) FT-IR spectrometer equipped with a MCT-A detector and a VeeMax (Pike Tech.) in a thin-layer configuration following the methodology illustrated in references 4 and 5. The glass spectroelectrochemical cell was coupled to a prismatic BaF_2 window beveled at 60°. BaF_2 window is used in order to work with a wider spectral region, since the cut-off of a BaF_2 is shifted to lower wavenumbers (ca. 900 cm^{-1}) relative to that of a CaF_2 prism (ca. 1100 cm^{-1}). [6] Although the solubility in aqueous media of BaF_2 is about two orders of magnitude higher than that of CaF_2, the low acidity of the solutions in this work and the high concentration of F^- hinder the dissolution of the window allowing acceptable times for carrying out the experiment. The experimental spectra

were collected at a resolution of 4 cm^{-1} employing the substractively normalized interfacial Fourier transform infrared reflection spectroscopy (SNIFTIRS) procedure,[7-9] in which 10 IR reflection spectra were collected at two pre-set potentials, a reference potential (E_{ref}) and a sample potential (E_{sample}) and repeated 3 times until 30 total interferograms were acquired for each of the two potentials. The resulting spectra are presented in absorbance units (a. u.) as $-\log(R/R_0)$, where R and R_0 represent the single-beam sample and reference reflectivity spectra, respectively. Therefore, positive-going bands correspond to the formation of species for the sample spectrum with respect to the reference spectra, while negative bands correspond to the consumption of species.

SUPPORTING INFORMATION

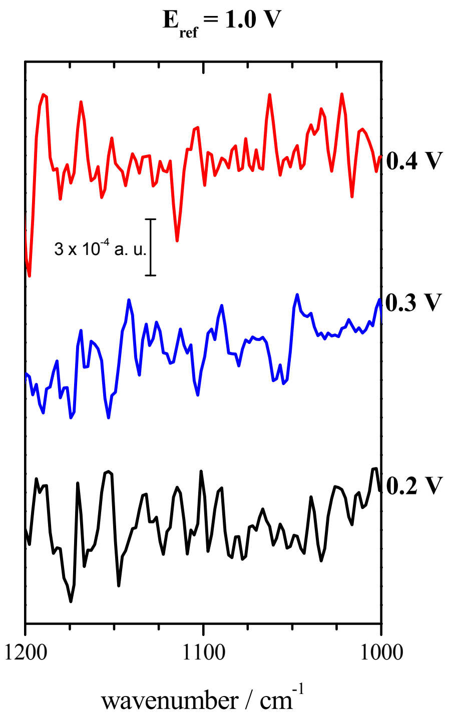

Fig. S1: In situ FT-IR spectra for Pt(111) in Ar-saturated $NaF/HClO_4$ mixture solution with pH = 5.5. Number of interferograms: 30; resolution: 4 cm^{-1}.

AUTHOR INFORMATION

Notes

‡(W.Ch.) Present address: Departamento de Química, Facultad de Ciencias, Pza. Misael Bañuelos s/n, 09001, Burgos, Spain

The authors declare no competing financial interests.

ACKNOWLEDGMENT

This work has been financially supported by MCINN (FEDER) (Spain) through project PID2019-105653GB-100.

REFERENCES

(1) Clavilier, J.; Faure, R.; Guinet, G.; Durand, R. Preparation of Monocrystalline Pt Microelectrodes and Electrochemical Study of the Plane Surfaces Cut in the Direction of the {111} and {110} Planes *J. Electroanal. Chem.* **1980**, *107*, 205-209.

(2) Clavilier, J. Flame-Annealing and Cleaning Technique. In *Interfacial Electrochemistry*; Wieckowski, A., Ed.; Marcel Dekker, Inc.: New York, 1999; pp 231-248.

(3) Korzeniewski, C.; Climent, V.; Feliu, J. M. Electrochemistry at Platinum Single Crystal Electrodes. In *Electroanalytical Chemistry: A Series of Advances*; Bard, A. J., Zoski, C., Eds.; CRC Press: Boca Raton, 2012; Vol. 24; pp 75-169.

(4) Iwasita, T.; Nart, F. C. In Situ Infrared Spectroscopy at Electrochemical Interfaces. *Prog. Surf. Sci.* **1997**, *55*, 271-340.

(5) Rodes, A.; Pérez, J. M.; Aldaz, A. Vibrational Spectroscopy. In *Handbook of Fuel Cells - Fundamentals, Technology and Applications*; Vielstich, W., Lamm, A., Gasteiger, H. A., Eds.; John Wiley & Sons, Ltd: Chichester, 2003; Vol. 2.

(6) Zamlynny, V.; Lipkowski, J. Quantitative SNIFTIRS and PM IRRAS of Organic Molecules at Electrode Surfaces. In *Diffraction and Spectroscopic Methods in Electrochemistry*; Alkire, R. C., Kolb, D. M., Lipkowski, J., Ross, P. N., Eds.; Wiley-VCH Verlag GmbH & Co. KGaA: Weinheim, 2006; pp 315-376.

(7) Iwasita, T.; Vielstich, W. New in-Situ IR Studies on Adsorption and Oxidation of Methanol on Platinum in Acidic Solution. *J. Electroanal. Chem.* **1988**, *250*, 451.

(8) Corrigan, D. S.; Weaver, M. J. Mechanisms of Formic Acid, Methanol, and Carbon Monoxide Electrooxidation at Platinum as Examined by Single Potential Alteration Infrared Spectroscopy. *J. Electroanal. Chem. Interfacial. Electrochem.* **1988**, *241*, 143-162.

(9) Cheuquepán, W.; Pérez, J. M.; Orts, J. M.; Rodes, A. Spectroelectrochemical and DFT Study of Thiourea Adsorption on Gold Electrodes in Acid Media. *J. Phys. Chem. C* **2014**, *118*, 19070-19084.
